# Molecular Signature of a Novel Alternanthera Yellow Vein Virus Variant Infecting the *Ageratum conyzoides* Weed in Oman

**DOI:** 10.3390/v15122381

**Published:** 2023-12-04

**Authors:** Muhammad Shafiq, Gabrijel Ondrasek, Abdullah Mohammed Al-Sadi, Muhammad Shafiq Shahid

**Affiliations:** 1 Department of Plant Sciences, College of Agricultural and Marine Sciences, Sultan Qaboos University, Al-Khoud, Muscat 123, Oman; 2 Faculty of Agriculture, University of Zagreb, Svetosimunska Cesta 25, 10000 Zagreb, Croatia

**Keywords:** Alternanthera yellow vein Oman virus, *Bemisia tabaci*, tomato leaf curl betasatellite, *Ageratum conyzoides*

## Abstract

Alternanthera yellow vein virus (AlYVV), a monopartite *begomovirus*, has been identified infecting a diverse range of crops and native plants in Pakistan, India, and China. However, distinctive yellow vein symptoms, characteristic of *begomovirus* infection, were observed on the *Ageratum conyzoides* weed in Oman, prompting a thorough genomic characterization in this study. The results unveiled a complete genome sequence of 2745 base pairs and an associated betasatellite spanning 1345 base pairs. In addition, Sequence Demarcation Tool analyses indicated the highest nucleotide identity of 92.8% with a previously reported AlYVV-[IN_abalpur_A_17:LC316182] strain, whereas the betasatellite exhibited a 99.8% nucleotide identity with isolates of tomato leaf curl betasatellite. Thus, our findings propose a novel AlYVV Oman virus (AlYVV-OM) variant, emphasizing the need for additional epidemiological surveillance to understand its prevalence and significance in Oman and the broader region. To effectively manage the spread of AlYVV-OM and minimize its potential harm to (agro)ecosystems, future research should focus on elucidating the genetic diversity of AlYVV-OM and its interactions with other begomoviruses.

## 1. Introduction

Circular Rep-encoding single-stranded (CRESS) DNA viruses, identified within the Cressdnaviricota phylum, encompass single-stranded DNA viruses encoding a replication-associated protein (Rep), believed to have a shared ancestral origin [1]. These viruses that infect plants fall into two families: *Geminiviridae* and *Nanoviridae* [2]. Among these, Begomoviruses stand as the largest genus among single-stranded DNA viruses, constituting 88% of the *Geminiviridae* family. They pose a significant threat to global crop yields, proficiently transmitted by the polyphagous whitefly vector, *Bemisia tabaci*, in a circulative manner, which comprises various biotypes [3]. This efficient vectorization extends across a wide range of hosts, spanning both cultivated and wild plant species [4,5]. Additionally, other species like *Trialeurodes ricini* and *Trialeurodes vaporariorum* have also been identified as potential transmitters of Begomoviruses [6]. Geminiviruses are divided into New-World (America) or Old-World (OW) (Australia, Asia, Mediterranean and Europe) viruses. According to the updated list of ICTV guidelines, the family *Geminiviridae* is divided into 14 genera (with 520 species) depending on (i) the host they infect, (ii) the insect vector which helps in transmission, and (iii) the genome organization. Among all genera, *Begomovirus* is the largest genus (with 445 known species), which infects a wide range of plant hosts, causing enormous economic losses to crops and ornamental plants globally [7]. Begomoviruses are further divided into two groups: (i) monopartite (having a single genome molecule, with the majority of them being associated with satellite molecules such as alphasatellites, betasatellites, and/or deltasatellites), and (ii) bipartite (having two genome molecules, known as DNA A and DNA B). The DNA A molecule of bipartite begomoviruses has a genome organization analogous to that of monopartite begomoviruses. Due to the small size of their genome (~2.7 kb), begomoviruses encode only a few proteins with diverse virulence functions. In general, the DNA-A component of both mono- and bipartite begomoviruses typically encode six proteins necessary for viral replication, movement, and the regulation of gene expression. Out of these two open reading frames (ORFs), V1 and V2 are located on the V-strand known as the virion strand, and four ORFs (C1–C4) are available on the C-strand known as the complimentary strand. The ORF V1 encodes for a coat protein (CP), V2 encodes for a pre-coat protein, C1 encodes for a replication-associated protein (Rep), C2 encodes for a transcriptional activation protein (TrAP), C3 encodes for a replication enhancer protein (REn), and C4 encodes for the C4 protein, which usually functions as a determinant of pathogenicity. The DNA-B molecule has two identified ORFs: one on the V-strand (BV1), which encodes for the nuclear shuttle protein (NSP), and the second on the C-strand (BC1), which encodes for the movement protein (MP). However, recent studies have identified an additional ORF named V3 in tomato yellow leaf curl virus (TYLCV). This ORF is suggested to play a role in full virus infection, potentially contributing to the virus’s ability to infect and propagate within host plants [8]. In addition, alphasatellites and betasatellites are crucial components for the majority of monopartite begomoviruses in the OW, each encoding a single recognized protein. Alphasatellites specifically code for the Rep protein, pivotal in the autonomous replication of satellites. However, betasatellites encode a solitaprotein on their C-strand, referred to as beta C1 (βC1), a well-established pathogenicity determinant, whose complex role has been thoroughly reviewed recently [9].

Begomoviruses in Oman have been the subject of limited diversity, epidemiological exploration, and gene function studies, with only a handful of reports documenting exotic strains [10,11,12]. In the present study, we aimed to fill this gap by investigating the introduction of a new *begomovirus*, AlYVV, infecting *Ageratum conyzoides* in Oman.

## 2. Material and Methods

### 2.1. Specimen Collection and DNA Extraction

**Figure 1:**
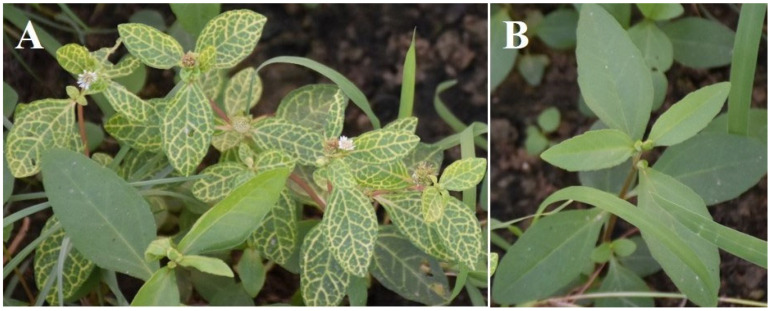
*Ageratum conyzoides* plant naturally infected by a bipartite AlYVV-OM, showing yellow vein symptoms (**A**), and asymptomatic plant (**B**).

During 2020, distinctive yellow vein symptoms indicative of *begomovirus* infection were observed on six *Ageratum conyzoides* plants with an incidence of 20–35%, which were collected from open field crops from Musandam (coordinates 26.1644° N 56.2426° E), Oman (Figure 1A). Newly emerging leaves, six symptomatic and three asymptomatic (Figure 1), were harvested, placed in a cold box, and transported to the plant biotechnology lab for a comprehensive genomic study. Subsequently, total genomic DNA extraction was conducted using CTAB protocols, following previously suggested procedures [13] with minor adjustments.

### 2.2. Amplification, Cloning, and Sequencing

**Figure 2:**
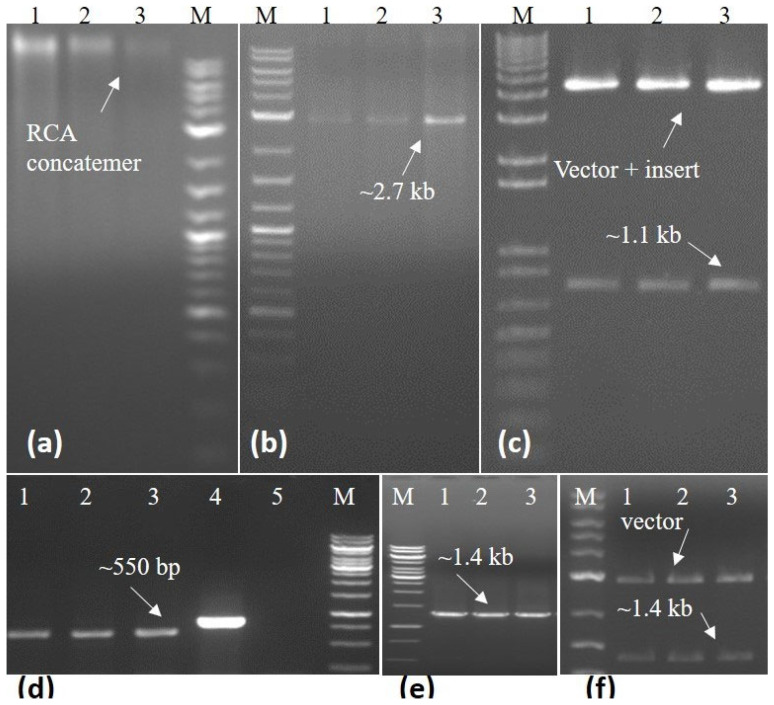
Three *Ageratum conyzoides* plants confirming positive detection in PCR were selected for further full-length amplification of begomovirus through RCA and betasatellite by specific primers. Amplification of RCA concatemers using Phi29 DNA polymerase enzyme from plants 1–3 (**a**). The monomer molecule of begomovirus was produced through restricted digestion using the *Bam*H1 enzyme for RCA from plants 1–3 (**b**). The production of recombinant begomovirus molecules from RCA restricted for plants 1–3 (**c**). Amplification of AlYVV-OM using PCR for plants 1–3 and lanes 4 and 5 served as the positive and negative controls, respectively (**d**). Amplification of tomato leaf curl betasatellite (ToLCB) through PCR (**e**). The insertion of ToLCB into a cloning vector amplified from plants 1–3 (**f**). “M” represents the 1 kb molecular marker employed to gauge the size of the DNA fragments in each case.

The isolated DNA was quantified and dilutions were prepared for use in PCR reaction using *begomovirus*-specific primers, amplifying an approximately 550 bp fragment of the CP gene in standardized PCR conditions [14] (Figure 2d). The full-length *begomovirus* genome was amplified in rolling circle amplification (RCA) using phi 29 DNA polymerase in the TempliPhi 100 Amplification Kit (GE Healthcare, Life Sciences, Piscataway, NJ, USA), following established protocols [15]. The concatemer of RCA products was then subjected to restriction fragment length polymorphism (RFLP) to release monomer molecules. Finally, utilizing the *Bam*H1 restriction enzyme, a full-length linear molecule of the begomovirus was generated and subsequently cloned into pGEM5Zf+ (Promega, Madison, WI, USA) (Figure 2a–c). Simultaneously, the betasatellite was amplified with betasatellite-specific primers and cloned into the pTZ57R/T cloning vector (Thermo Fisher Scientific, Waltham, MA, USA) (Figure 2e,f).

### 2.3. Bioinformatic Analysis

After the cloning and confirmation of full-length clones for begomovirus (*n* = 3) and betasatellite (*n* = 3), one begomovirus and betasatellite from each plant were subjected to complete Sanger sequencing at Macrogen Inc. (Seoul, Republic of Korea). Multiple sequence contigs were received and assembled using SeqMan, built in the DNAstar Lasergene package (Madison, WI, USA). Initial genome identification was performed in a BLASTn search (https://blast.ncbi.nlm.nih.gov/Blast.cgi, accessed on 16 December 2021), and highly similar sequences were retrieved from GenBank for pairwise sequence analysis using the sequence demarcation tool [16]. Multiple sequence alignments were performed using MUSCLE, and phylogenetic tree was constructed in MEGAX using the neighbor-joining algorithm, selecting the best-fit Kimura-2 parameter with 1000 bootstrap values [17]. Potential recombination events were verified using the RDP4 program with default settings and a cut-off value of *p* ≤ 0.05 [18]. Predicted ORFs were identified using ORF Finder (https://www.ncbi.nlm.nih.gov/orffinder/, accessed on 5 January 2022).

### 2.4. Population Study

To identify the genetic diversity of the virus population, different parameters of the DnaSP (v. 6.12) program were used [19,20]. The multiple aligned sequences were studied for the entire segregating sites (s), the average number of nucleotide differences between sequences (kt), the total number of mutations (Eta), the sequence nucleotide diversity (π), and Watterson’s estimate of the population mutation rate based on the total number of segregating sites (θ − w), and the total number of mutations (θ–η) were also analyzed along with the number of haplotypes (h) and the haplotype diversity (Hd) [21].

## 3. Results

### 3.1. Alternanthera Yellow Vein Oman Virus Is Associated with Ageratum conyzoides

The presence of the monopartite begomovirus in symptomatic *A. conyzoides* was confirmed through PCR analysis. Notably, six symptomatic samples exhibited bands of approximately 700 base pairs when subjected to detection primers. None of the healthy plants exhibited any amplification of the PCR product. Utilizing Phi29 DNA polymerase and hexamer primers in RCA amplification from three samples resulted in the generation of concatemers composed of circular DNA molecules. Subsequent RFLP analysis, employing the *Bam*H1 restriction enzyme, revealed that the monomer molecule of the expected begomovirus might be the causative agent in the infected *A. conyzoides* plants.

**Figure 3:**
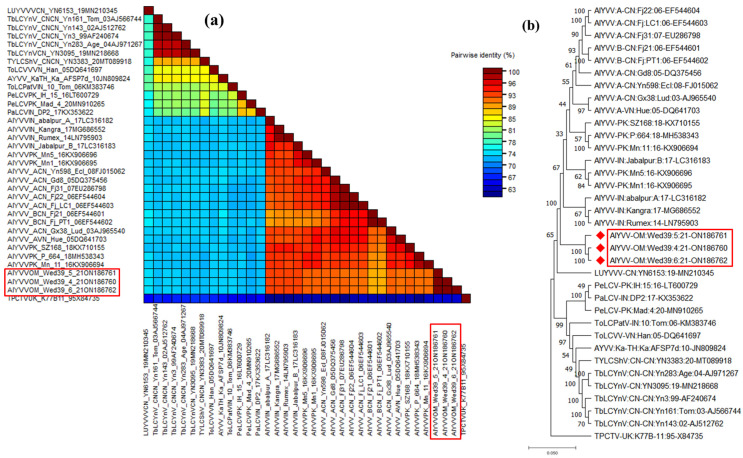
Pairwise sequence analysis using sequence demarcation tool (SDT V1.2) of AlYVV-OM isolates highlighted in red boxes identified from this study and used for analysis (**a**), and phylogenetic dendrograms based on complete nucleotide sequences of DNA A of AlYVV-OM genome components (**b**). To calculate mutation distances, vertical and horizontal branches are arbitrary and proportional, respectively. The virus tree was arbitrarily rooted on the sequence of tomato curly top virus. The begomovirus acronyms used were ludwigia yellow vein Vietnam virus (LUYVVV), tobacco leaf curl Yunnan virus (TbLCYNV), tomato leaf curl Vietnam virus (TOLCVV), ageratum yellow vein virus (AYVV), tomato leaf curl Patna virus isolate (ToLCPV), pedilanthus leaf curl virus (PeLCV), papaya leaf curl virus (PaLCV), and alternanthera yellow vein virus (AYVV). A diverse DNA sequence from the tomato pseudo-curly top virus (TPCTV) was employed for comparative purposes.

Three full-length begomoviruses, each derived from a different plant, were sequenced using the Sanger method, and all sequencing contigs were assembled to produce a predictable begomovirus genome. The complete genome sequence of this begomovirus spanned 2791 bp and exhibited the typical genome orientation observed in OW monopartite begomoviruses with six ORFs (V2 and CP in the virion-sense and Rep, TrAP, Ren, and C4 protein in the complementary sense; Table 1). To ensure accuracy, any ambiguities in the complete genome sequences were resolved before submission to the NCBI GenBank, currently accessible under numbers ON186760-ON186762. The pairwise sequence analysis using the STD tool revealed the highest nucleotide identity at 92.8% with an isolate of alternanthera yellow vein virus (AlYVV) AlYVV-[IN: abalpur:A:17] (LC316182), which infects *Alternanthera sessilis* in India [22]. Subsequently, nucleotide identities ranged from 92.5% to 90.7% with other AlYVV isolates, while a distinct range of 72.2% to 74% of nucleotide identity was observed with various other begomovirus species (Figure 3a and Table 2). The identified isolate undergoes scrutiny as a novel variant, AlYVV-OM, which specifically infects the *A. conyzoides* weed in Oman [23]. The phylogenetic tree further reinforced this conclusion by clustering Omani isolates (AlYVV-OM-wed39-4, AlYVV-OM-wed39-5, and AlYVV-OM-wed39-6) with AlYVV isolates described earlier in the same group supported by 1000 bootstrap values (Figure 3b).

Population structure analyses, performed to evaluate the intensity of genetic variability among the *begomovirus* sequences under study, confirmed the total number of polymorphic segregating sites (S = 1309), with 681 InDel sites, the haplotype diversity (hd = 0.998), the total mutation (Eta = 2285), the average number of nt. differences (Kt = 460), and the nucleotide diversity (π = 0.17) for AlYVV-OM datasets (Table 3). Similarly, the analysis performed for ORF datasets revealed the maximum number of polymorphic sites (s) and number of mutations (η) in AC1 (Rep) and AV1 (CP) genes with nucleotide diversity π = 0.1. Moreover, for betasatellites, the total number of polymorphic sites (s) was 834, with a total number of mutations (η) of 1482 and a nucleotide diversity (π) of 0.30509 (π = 0.3), which were also very high. However, the obtained maximum π value explains the non-random distribution of nucleotides throughout viral and sub-viral genome regions, which significantly contributes to a high degree of genetic variability. Therefore, this estimation suggests the presence of highly diverse populations, within and among populations.

Furthermore, strong evidence of population genetic diversity is evident through both the total number of haplotypes (H = 33) and the high haplotype diversity (Hd = 0.99) (Table 3). Simultaneously, within the ORF datasets, the values for H and Hd span ranges of 31–42 and 0.96 to 0.99, respectively. This result underscores the varying contribution of genes to DNA polymorphism. Similarly, for betasatellites, the values for H and Hd were 17 and 1.000, respectively, indicating a low level of sequence divergence, but a notable frequency of unique mutations (Table 3).

### 3.2. Tomato Leaf Curl Betasatellite Associated with the Disease

Three full-length clones (wed39-7, wed39-8, and wed39-9), each spanning 1375 base pairs, were identified and subsequently submitted to NCBI GenBank under the accession numbers ON186763-ON186765.

**Figure 4:**
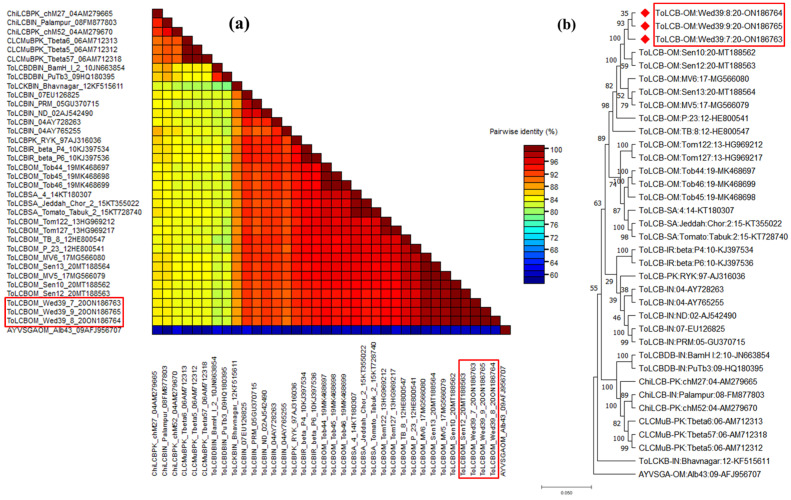
Pairwise sequence analysis using sequence demarcation tool (SDT V1.2) of ToLCB isolates highlighted in red boxes identified from this study and used for analysis (**a**), and phylogenetic dendrograms based on complete nucleotide sequences of ToLCB genome components (**b**). To calculate mutation distances, vertical and horizontal branches are arbitrary and proportional, respectively. The betasatellite tree was arbitrarily rooted on the sequence of ageratum yellow vein Singapore alphasatellite. The betasatellite acronyms used are chilli leaf curl betasatellite (ChiLCB), cotton leaf curl Multan betasatellite (CLCMuB), and tomato leaf curl betasatellite (ToLCB). A varied DNA sequence from the ageratum yellow vein Singapore alphasatellite (AYVSGA) was utilized for comparative analysis.

These isolates exhibit a high degree of similarity, presenting betasatellite genomes characterized by a single predicted beta C1 ORF on the virion-sense strand. Additionally, a notable feature includes a region rich in adenine sequences (A-rich) and a conserved satellite region (SCR), a commonality observed across all betasatellites. In pairwise sequence analysis using SDT, these isolates displayed a maximum nt. identity at 99.8–99.9% with tomato leaf curl betasatellite ToLCB- [OM: Sen10:20] (MT188562), previously reported in Oman [14]. Subsequently, they exhibited identities of 88.2%, 83.5%, and 84.7% with ToLCKB, CLCMuB, and ChiLCB isolates, respectively (Figure 4a). In the phylogenetic analysis, these isolates formed a distinct cluster with other ToLCB isolates, supported by 1000 bootstrap values, confirming their association with ToLCB (Figure 4b). According to the ICTV guidelines set for betasatellite classification (>79% identity), the identified clones are recognized as a novel isolate of ToLCB, linked with AlYVV-OM, and infecting a new host—*A. conyzoides* in Oman.

### 3.3. Recombination Analysis

To explore the potential role of recombination in AlYVV-OM, a comprehensive recombination analysis was conducted using complete genome sequences of AlYVV-OM and 100 isolates of other begomoviruses retrieved from GenBank under default settings. Although the predicted ORFs of AlYVV-OM did not reveal any variable sequence identity with the corresponding ORFs of other begomoviruses, and therefore indicating that AlYVV-OM is not a recombinant virus, it is noteworthy that AlYVV-OM has been involved as a major/minor parent, potentially contributing to recombination events that might influence the evolution of other begomoviruses. Therefore, in the Recombination Detection Program (RDP) analysis, AlYVV-OM was identified as a significant contributor in the recombination of AlYVV (LC316182), serving as a major parent according to at least four algorithms: Maxchi (1.38 × 10^−6^), Chimaera (3.54 × 10^−5^), SiSscan (1.26 × 10^−24^), and 3Seq (2.91 × 10^−6^). In addition, for ageratum yellow vein China virus (KU954382), AlYVV-OM was recognized as a minor parent, with GENECONV (4.34 × 10^−4^) and SiScan (1.99 × 10^−73^) algorithms considered as reliable indicators of this involvement.

### 3.4. Population Study

Virus population analysis was applied to calculate the degree of genetic variability (>0.08) within and among populations (Table 3). However, we observed a total of 1309 polymorphic sites (s), encompassing 2285 mutations (η), and a nucleotide diversity of 0.17 (π = 0.1) for AlYVV-OM. Likewise, in the case of betasatellites, we identified a total of 900 polymorphic sites (s), with a noteworthy 1200 mutations (η), resulting in a substantial nucleotide diversity (π) of 0.12 (π = 0.3). However, the obtained maximum π value explains the non-random distribution of nucleotides throughout viral and sub-viral genome regions, which significantly contributes to a high degree of genetic variability. Therefore, the estimation suggests diverse populations within and among populations. In addition, the genetic diversity within and among populations was also determined by the number of haplotypes (H) and haplotype diversity (Hd). Therefore, using DnaSP software (v. 6.0) [20], analysis was performed for sequence datasets that contained a haplotype distribution among reference DNA sequences of the *begomovirus* population, and we found a total number of haplotypes (H) of 33, where its haplotype diversity was identified as close to 1, i.e., Hd = 0.99. Therefore, the overall result explains the low level of sequence divergence but the high frequency of unique mutations (Table 4).

## 4. Discussion

Over the past two decades, various factors such as the transport of infected materials, vector population dynamics, and biological and environmental changes have played a vital role in the emergence of new *begomovirus* species and/or strains in different geographical areas, infecting diverse host plant species [34]. Consequently, previously unrecognized hosts, including crops, ornamental plants, weeds, and trees, have become susceptible to different begomoviruses. Despite limited studies reporting the incidence of AlYVV, we present the first-ever documentation of a novel variant, AlYVV-OM, in Oman. The occurrence of this virus has been observed in limited geographical areas and the complete genome sequence of AlYVV-OM was identified from the *Ageratum conyzoides* host.

Comparison with other publicly available AlYVV isolates (12 sequences) revealed that AlYVVOM exhibited the highest nucleotide identity (92.5%) with an Indian isolate, highlighting its close relation to an Indian isolate among the publicly available AlYVV sequences. AlYVV was initially discovered in China in 2005, primarily infecting *Alternanthera philoxeroides,* sharing a notable genomic similarity and exhibiting the highest nucleotide identity with ageratum yellow vein virus AYVV [33]. Its subsequent appearances in Vietnam and Pakistan during 2008 and 2010, respectively, associated the virus with diverse DNA satellites, encompassing ageratum yellow leaf curl betasatellite, cotton leaf curl Multan betasatellite (CLCuMuB), potato leaf curl alphasatellite (PotLCuA), and hi-biscus leaf curl alphasatellite (HLCuA) [35]. Furthermore, a report in India highlighted its presence alongside CLCuMuB, infecting Picrorhiza kurroa plants [24] (Table 2). This intercontinental prevalence underscores the adaptability and broad host range of AlYVV.

The majority of begomoviruses prevalent in South Asia (China, India, and Pakistan) differ from those in the Arabian Peninsula, owing to the natural sea barrier. However, the very close genetic relationship between AlYVV-OM and Indian isolates suggests a likely origin from India, with the possibility that trade via sea or air routes facilitated the distribution of this virus into Oman. Additionally, it is noteworthy that AlYVV identified in China was not reported to be accompanied by betasatellites. In contrast, Ha et al. [36] reported an AlYVV isolate from Vietnam to be associated with alternanthera yellow vein betasatellite. This suggests that AlYVV has the capability to infect plants in the field, regardless of the presence or absence of a betasatellite [37]. Notably, the association of betasatellites may enhance begomoviruses’ ability to overcome host defense responses, broaden host ranges, and indirectly impact in planta virus accumulation [38]. Although betasatellite molecules have no precise nucleotide sequence identity with the helper (mono-or-bipartite) begomoviruses, they exhibit conserved nonanucleotide (TAATATT/AC) sequences [39]. Betasatellites are very adaptable in their trans-replication with diverse begomoviruses, including other members of the *Geminiviridae* family. The Rep protein in the majority of begomoviruses supports the replication of different betasatellites, even in cases where they lack cognate-related virus iteron sequences [40]. Thus, it is assumed that virus–betasatellite interactions are non-specific and related to trans-replication, long-distance movement, and transmission [41]. The particular sequences in betasatellites mimic the iterons for the rep binding, which illustrates its indiscriminate replicative nature [42]. Due to a similar purpose, it is very likely that AlYVV can effectively trans-replicate ToLCB. The vector (whitefly) population and its polyphagous nature also contributed to the evolution of these begomoviruses.

The identification of various begomoviruses and betasatellites in *Ageratum conyzoides* underscores the potential importance of weeds as alternate hosts, acting as reservoirs for the emergence of diverse *begomovirus* species worldwide. Thus, we hypothesize that AlYVV-OM may be an ancient virus that has evolved over time within *Ageratum conyzoides*, possessing the capability to interact with ToLCB. Furthermore, our findings indicate that ToLCB can form associations with AlYVV-OM, potentially facilitating its interaction with various begomoviruses, such as TYLCV, a globally distributed begomovirus frequently observed in Oman. The current study also forms a foundation for further studies on the epidemiology and genetic diversity of AlYVV-OM in Oman and the wider region.

## 5. Conclusions

The complete genomes of novel AlYVV-OM and ToLCB were meticulously examined within the weed host *Ageratum conyzoides*, offering valuable insights into their epidemiology and emphasizing the importance of comprehending their population structure. Considering the potential evolution of diverse begomoviral strains within specific geographical areas and their possible spread to other regions through various transmission routes, it becomes imperative to implement protective measures. To curtail the transmission of these pathogens, it is highly recommended to regulate trade products through enhanced phytosanitary measures and establish facilities for the early detection of viruses. Implementing such measures is crucial for preventing and controlling the spread of viruses across regions, thereby protecting against potential harm and ensuring the long-term sustainability of (agro)ecosystems.

## Figures and Tables

**Table 1:** Features of alternanthera yellow vein Oman virus and tomato leaf curl betasatellite isolated from *Ageratum conyzoides* plants.

			Alternanthera Yellow Vein Virus Position of Genes (Coordinates)/No. of Amino Acids (Predicted Coding Capacity in kDa)							Tomato Leaf Curl Betasatellite		
Isolate	Acc. No.	Size (nt)	CP	V2	Rep	TrAP	REn	C4	Satellite	Acc. No.	Size (nt)	Position of BC1 Gene (Coordinates)/No. of Amino Acids (Predicted Coding Capacity in kDa)
Wed39-4	ON186760	2745	314–1084 256	154–501 115	1533–2618 362	1226–1630 134	1081–1485 134	2171–2467 98	Wed39-7	ON186763	1360	189–545 118 (13)
Wed39-5	ON186761	2747	314–1084 256	154–501 115	1533–2618 362	1226–1630 134	1081–1485 134	2171–2467 98	Wed39-8	ON186764	1360	189–545 118 (13)
Wed39-6	ON186762	2747	314–1084 256	154–501 115	1533–2618 361	1226–1630 134	1081–1485 134	2171–2467 98	Wed39-9	ON186765	1360	189–545 118 (13)

**Table 2:** Percent nucleotide sequence identities of alternanthera yellow vein Oman virus (AlYVV-OM) with other related begomoviruses.

Virus Name *	Isolate	Host Species	Family	Region/Country	GenBank Accession No.	Percent Nucleotide Sequence Identity
LUYVV	YN6153	*Ludwigia linifolia*	*Onagraceae*	China	MN210345	74%
TbLCYnV	Y161	tomato	*Solanaceae*	China	AJ566744	73.1%
TbLCYnV	Y143	tobacco	*Solanaceae*	China	AJ512762	73.2%
TbLCYnV	Y3	tobacco	*Solanaceae*	China	AF240674	73%
TbLCYnV	Y283	*Ageratum conyzoides*	*Asteraceae*	China	AJ971267	73.2%
TbLCYnVCN	YN3095	*Ageratum conyzoides*	*Asteraceae*	China	MN218668_	73.2%
TYLCShV	YN3383	cowpea	*Fabaceae*	China	MT089918	73.1%
TYLCVNV	Vt-6	tomato	*Solanaceae*	Vietnam	DQ641697	73.2%
AYVV	AFSP7d	*Sauropus androgynus*	*Phyllanthaceae*	Thailand	JN809824	73.3%
ToLCPatV	ToLCPatV	tomato	*Solanaceae*	India	KM383746	73.3%
PeLCV	Pk2	*Raphanus sativus*	*Brassicaceae*	Pakistan	LT600729	72.5%
PeLCV	Mad-4	soybean	*Fabaceae*	Pakistan	MN910265	72.2%
PeLCV	DP2	papaya	*Caricaceae*	India	KX353622	75.5%
AlYVV	Jabalpur_A	*Alternanthera sessilis*	*Amaranthaceae*	India	LC316182	92.8%
AlYVV	Kangra	*Synedrella* sp.	*Asteraceae*	India	MG686552	92.6%
AlYVV	Rumex	*Rumex nepalensis*	*Polygonaceae*	India	LN795903	92.5%
AlYVV	Jabalpur_B	*Alternanthera sessilis*	*Amaranthaceae*	India	LC316183	92.5%
AlYVV	Mn5	*Eclipta prostrata*	*Asteraceae*	Pakistan	KX906696	92.4%
AlYVV	Mn1	*Eclipta prostrata*	*Asteraceae*	Pakistan	KX906695	91.6%
AlYVV	YN598	*Eclipta prostrata*	*Asteraceae*	China	FJ015062	91.1%
AlYVV	G8	*Eclipta prostrata*	*Asteraceae*	China	DQ375456	91.6%
AlYVV	F31	*Eclipta prostrata*	*Asteraceae*	China	EU286798	91.1%

* ageratum yellow vein virus, alternanthera yellow vein virus, papaya yellow leaf curl virus, pedilanthus leaf curl virus, tobacco leaf curl Yunnan virus, tomato leaf curl Patna virus, tomato yellow leaf curl Shuangbai virus, tomato yellow leaf curl Vietnam virus.

**Table 3:** Nucleotide diversity of alternanthera yellow vein Oman virus (AlYVV-OM) and ToLCB.

Virus	No of Seq	H	Hd	InDel Sites	S	Eta	Kt	π
AlYVOMV	34	33	0.998	681	1309	2285	460	0.17
ToLCB	35	33	0.996	283	900	1200	138	0.12

H; haplotypes, Hd; haplotype diversity, InDel sites; total number of InDel sites, S; number of polymorphic segregating sites, Eta; total number of mutations, Kt; average number of nucleotide differences, π; nucleotide diversity.

**Table 4:** Geographical distribution of alternanthera yellow vein virus and its host range.

Virus Name	Isolate Name	Host Species	Family	Country or Region	Reference
Alternanthera yellow vein virus	AlYVV	*Alternanthera sessilis*	*Amaranthaceae*	India	[22]
Alternanthera yellow vein virus	AlYVV	*Picrorhiza kurroa*	*Plantaginaceae*	India	[24]
Alternanthera yellow vein virus	AlYVV	*Eclipta prostrata*	*Asteraceae*	Pakistan	[25]
Alternanthera yellow vein virus	AlYVV	*Eclipta prostrata*	*Asteraceae*	Pakistan	[26]
Alternanthera yellow vein virus	AlYVV	*Eclipta prostrata*	*Asteraceae*	Pakistan	[27]
Alternanthera yellow vein virus	AlYVV	*Eclipta prostrata*	*Asteraceae*	China	[28]
Alternanthera yellow vein virus	AlYVV	*Sonchus arvensis*	*Asteraceae*	Pakistan	[29]
Alternanthera yellow vein virus	AlYVV	*Eclipta prostrata*	*Asteraceae*	China	[30]
Alternanthera yellow vein virus	AlYVV	*Eclipta prostrate* *(*L.*)*	*Compositae*	China	[31]
Ludwigia yellow vein virus	LUYVV	*Ludwigia hyssopifolia*	*Onagraceae*	China	[32]
Alternanthera yellow vein virus	AlYVV	*Alternanthera philoxeroides*	*Amaranthaceae*	China	[33]

## Data Availability

The data generated during this research have been included in this published article.

## References

[1] Krupovic M., Varsani A., Kazlauskas D., Breitbart M., Delwart E., Rosario K., Zerbini F.M. (2020). Cressdnaviricota: A virus phylum unifying seven families of rep-encoding viruses with single-stranded, circular DNA genomes. J. Virol..

[2] Dolja V.V., Krupovic M., Koonin E.V. (2020). Deep roots and splendid boughs of the global plant virome. Annu. Rev. Phytopathol..

[3] Gilbertson R.L., Batuman O., Webster C.G., Adkins S. (2015). Role of the Insect Supervectors Bemisia tabaci and Frankliniella occidentalis in the Emergence and Global Spread of Plant Viruses. Annu. Rev. Virol..

[4] Zerbini F.M., Briddon R.W., Idris A., Martin D.P., Moriones E., Navas-Castillo J., Ictv Report C. (2017). ICTV Virus Taxonomy Profile: Geminiviridae. J. Gen. Virol..

[5] Fiallo-Olive E., Navas-Castillo J. (2020). Molecular and Biological Characterization of a New World Mono-/Bipartite Begomovirus/Deltasatellite Complex Infecting Corchorus siliquosus. Front. Microbiol..

[6] Idriss M., Abdallah N., Aref N., Haridy G., Madkour M. (1997). Biotypes of the castor bean whitefly *Trialeurodes ricini* (Misra) (Hom., Aleyrodidae) in Egypt: Biochemical characterization and efficiency of geminivirus transmission. J. Appl. Entomol..

[7] Walker J., Siddell S.G., Lefkowitz E.J., Mushegian A.R., Adriaenssens E.M., Alfenas-Zerbini P., Zerbini F.M. (2021). Changes to virus taxonomy and to the International Code of Virus Classification and Nomenclature ratified by the International Committee on Taxonomy of Viruses (2021). Arch. Virol..

[8] Gong P., Tan H., Zhao S., Li H., Liu H., Ma Y., Zhou X. (2021). Geminiviruses encode additional small proteins with specific subcellular localizations and virulence function. Nat. Commun..

[9] Gnanasekaran P., Kishorekumar R., Bhattacharyya D., Vinoth Kumar R., Chakraborty S. (2019). Multifaceted role of geminivirus associated betasatellite in pathogenesis. Mol. Plant Pathol..

[10] Shahid M.S., Shafiq M., Ilyas M., Raza A., Al-Sadrani M.N., Al-Sadi A.M., Briddon R.W. (2019). Frequent occurrence of Mungbean yellow mosaic India virus in tomato leaf curl disease affected tomato in Oman. Sci. Rep..

[11] Al Shihi A.A., Al Sadi A.M., Deadman M., Briddon R.W., Shahid M.S. (2018). Identification of a distinct strain of Cotton leaf curl Gezira virus infecting tomato in Oman. J. Phytopathol..

[12] Shahid M.S., Al-Sulaimani H., Al-Sadi A.M. (2020). Squash Leaf Curl Virus: A New World Bipartite Begomovirus Threatening Squash Production in Oman. Plant Dis..

[13] Porebski S., Bailey L.G., Baum B.R. (1997). Modification of a CTAB DNA extraction protocol for plants containing high polysaccharide and polyphenol components. Plant Mol. Biol. Report..

[14] Al-Mabsli S.S., Al-Wahaibi A.K., Al-Sadi A.M., Shahid M.S. (2021). Association of a monopartite begomovirus and associated betasatellite with yellow vein disease of a weed host, *Senna italica* Mill. in Oman. Virusdisease.

[15] Haible D., Kober S., Jeske H. (2006). Rolling circle amplification revolutionizes diagnosis and genomics of geminiviruses. J. Virol. Methods.

[16] Muhire B.M., Varsani A., Martin D.P. (2014). SDT: A virus classification tool based on pairwise sequence alignment and identity calculation. PLoS ONE.

[17] Kumar S., Stecher G., Tamura K. (2016). MEGA7: Molecular evolutionary genetics analysis version 7.0 for bigger datasets. Mol. Biol. Evol..

[18] Martin D.P., Murrell B., Golden M., Khoosal A., Muhire B. (2015). RDP4: Detection and analysis of recombination patterns in virus genomes. Virus Evol..

[19] Lima A.T.M., Silva J.C.F., Silva F.N., Castillo-Urquiza G.P., Silva F.F., Seah Y.M., Zerbini F.M. (2017). The diversification of begomovirus populations is predominantly driven by mutational dynamics. Virus Evol..

[20] Rozas J., Ferrer-Mata A., Sánchez-Delbarrio J.C., Guirao-Rico S., Librado P., Ramos-Onsins S.E., Sánchez-Gracia A. (2017). DnaSP 6: DNA sequence polymorphism analysis of large data sets. Mol. Biol. Evol..

[21] Shafiq M., Sattar M.N., Shahid M.S., Al-Sadi A.M., Briddon R.W. (2021). Interaction of watermelon chlorotic stunt virus with satellites. Australas. PlantPathol..

[22] Marabi R.S., Das S.B., Tripathi N., Wada T., Noda H. (2021). Identification of begomoviruses from legume crop and weed plants and viruliferous status of the whitefly Bemisia tabaci in Central India. Curr. Sci..

[23] Brown J.K., Zerbini F.M., Navas-Castillo J., Moriones E., Ramos-Sobrinho R., Silva J.C., Varsani A. (2015). Revision of Begomovirus taxonomy based on pairwise sequence comparisons. Arch. Virol..

[24] Sharma D., Kulshreshtha A., Kumar R., Hallan V. (2019). First report of natural infection of alternanthera yellow vein virus and cotton leaf curl Multan betasatellite on a new host *Picrorhiza kurroa*, an important endangered medicinal herb. J. Plant Pathol..

[25] Murtaza G., Mubin M., Nawaz-Ul-Rehman M.S., Amrao L. (2018). Genetic analysis of alternanthera yellow vein virus (Ayvv) infecting *Eclipta prostrata* plant in Pakistan. Pak. J. Agric. Sci..

[26] Nawaz-Ul-Rehman M., Liaqat I., Nahid N., Saleem F., Alkahtani S., Al Qahtani A., Mubin M. (2022). Alternanthera yellow vein virus (AYVV); A betasatellite independent begomovirus infecting *Sonchus palustris* in Pakistan. Braz. J.Biol..

[27] Zaidi S.S., Shakir S., Farooq M., Amin I., Mansoor S. (2017). First report of Alternanthera yellow vein virus from *Eclipta prostrata* in Pakistan. Plant Dis..

[28] Zhang J., Jia S.P., Yang C.X., Liu Z., Wu Z.J. (2015). Detection and molecular characterization of three begomoviruses associated with yellow vein disease of *Eclipta prostrata* in fujian, China. J. Plant Pathol..

[29] Mubin M., Shahid M.S., Tahir M.N., Briddon R.W., Mansoor S. (2010). Characterization of begomovirus components from a weed suggests that begomoviruses may associate with multiple distinct DNA satellites. Virus Genes.

[30] Ding M., Yang L., Zhao Z.W., Zhang Z.K. (2009). First report of alternanthera yellow vein virus in *Eclipta prostrata* in China. J. Plant Pathol..

[31] He Z.F., Mao M.J., Yu H., Wang X.M., Li H.P. (2008). First report of a strain of Alternanthera yellow vein virus infecting *Eclipta prostrate* (L.) L. (compositae) in China. J. Phytopathol..

[32] Huang J.F., Jiang T., Zhou X.P. (2006). Molecular characterization of begomoviruses infecting *Ludwigia hyssopifolia*. J. Plant Pathol..

[33] Guo X., Zhou X. (2005). Molecular characterization of *Alternanthera* yellow vein virus: A new *Begomovirus* species infecting *Alternanthera philoxeroides*. J. Phytopathol..

[34] Rojas M.R., Hagen C., Lucas W.J., Gilbertson R.L. (2005). Exploiting chinks in the plant’s armor: Evolution and emergence of geminiviruses. Annu. Rev. Phytopathol..

[35] Idris A.M., Shahid M.S., Briddon R.W., Khan A., Zhu J.-K., Brown J.K. (2011). An unusual alphasatellite associated with monopartite begomoviruses attenuates symptoms and reduces betasatellite accumulation. J. Gen.Virol..

[36] Ha C., Coombs S., Revill P., Harding R., Vu M., Dale J. (2008). Molecular characterization of begomoviruses and DNA satellites from Vietnam: Additional evidence that the New World geminiviruses were present in the Old World prior to continental separation. J. Gen. Virol..

[37] Briddon R.W., Brown J.K., Moriones E., Stanley J., Zerbini M., Zhou X., Fauquet C.M. (2008). Recommendations for the classification and nomenclature of the DNA-beta satellites of begomoviruses. Arch. Virol..

[38] Briddon R.W., Markham G. (2001). Complementation of bipartite begomovirus movement functions by topocuviruses and curtoviruses. Arch. Virol..

[39] Hanley-Bowdoin L., Settlage S.B., Orozco B.M., Nagar S., Robertson D. (1999). Geminiviruses: Models for plant DNA replication, transcription, and cell cycle regulation. Crit. Rev. Plant Sci..

[40] Kharazmi S., Behjatnia S.A., Hamzehzarghani H., Niazi A. (2012). Cotton leaf curl *Multan betasatellite* as a plant gene delivery vector trans-activated by taxonomically diverse geminiviruses. Arch. Virol..

[41] Saunders K. (2008). Analysis of geminivirus DNA replication by 2-D gel. Methods Mol. Biol..

[42] Nawaz-Ul-Rehman M.S., Nahid N., Mansoor S., Briddon R.W., Fauquet C.M. (2010). Post-transcriptional gene silencing suppressor activity of two non-pathogenic alphasatellites associated with a begomovirus. Virology.

